# Compulsory community treatment orders (CTOs): recent research and future directions

**DOI:** 10.1192/bjo.2023.71

**Published:** 2023-05-22

**Authors:** Ben Beaglehole, Matthew Tennant

**Affiliations:** Department of Psychological Medicine, Christchurch School of Medicine, University of Otago, Christchurch, New Zealand

**Keywords:** Compulsory treatment order, CTO, coercion, evidence, outcomes

## Abstract

Compulsory community treatment orders (CTOs) are controversial because the right to refuse treatment is overridden, even when patients may not be acutely unwell. Scrutiny of outcomes associated with CTOs is therefore required. This editorial provides an overview of the evidence for CTOs. It also discusses recent papers reporting outcomes associated with CTOs and makes recommendations for researchers and clinicians to consider.

Compulsory community treatment orders (CTOs) were developed in response to deinstitutionalisation and the assumption there would be challenges providing care for people with serious mental illness in the community. CTOs require individuals to accept psychiatric treatment in the community with a key goal of reducing psychiatric admissions. Broader treatment goals, such as rapid access to psychiatric care and greater family involvement, are also considered by clinicians. CTOs are typically made when there are serious concerns about a patient's risks to self or others. Increasingly, for CTOs to be enacted mental health legislation also requires that the patient lack capacity.

CTOs are controversial because treatment may be enforced in the absence of active mental illness without the requirement for consent. An individual's right to autonomy over health decisions is often pitted against the desires of family or the need for community safety. However, risk assessments are criticised because it is difficult to predict rare outcomes such as suicide or homicide. Despite this, coercive treatment of patients perceived to be ‘high risk’ occurs, with a view to preventing suicides and violence. CTOs also cause unintended harm. Patients find them stigmatising, they may experience adverse drug reactions and coercive treatment may discourage help-seeking.^1^ Consequently, if we are to continue using CTOs it is important to understand the range of outcomes associated with their use.

## The evidence base

### Randomised controlled trials

Only three randomised controlled trials (RCTs) evaluating CTOs have been completed. This is understandable given the challenges of ensuring informed consent from patients subject to them and randomising patients into non-CTO arms when they are at serious risk of harm to self or others. Each of the RCTs evaluating CTOs has limitations, including the exclusion of certain patients from study entry,^2,3^ study sample attrition^4^ and the confounding effect of providing enhanced care as part of the study design.^4^ A Cochrane review concluded that the quality of evidence for the RCTs was low to moderate and that CTOs did not result in clear differences for the majority of outcomes delivered.^5^

### Cohort studies

Cohort studies provide an alternative means of assessing the outcomes of CTOs. Studies of this type use a ‘before and after’ CTO design or compare individuals discharged on a CTO following a compulsory admission with a matched control group discharged under voluntary status. Barnett et al reported that ‘before and after’ studies were associated with a large reduction in admission frequency whereas this was not the case for the matched control studies.^6^ The ‘before and after’ design is vulnerable to confounding influences, including regression to the mean and maturation effects. Although the use of matched controls allows the influence of time and the natural history of the psychiatric illness to be taken into account, we argue that matching controls on the basis of demographic variables fails to take into account intangible factors such as lack of insight and refusal to accept treatment (variables that are key to the decision to commence a CTO).^7^ We therefore completed a series of studies evaluating outcomes associated with CTOs using a ‘within-persons’ design, comparing outcomes on and off CTOs (as opposed to using a control group) for a national cohort placed on CTOs over a 10-year period.^7–9^ Individuals could be on multiple CTOs over this period. This approach allowed key outcomes associated with CTOs to be compared while they are enacted rather than at a distant time period. We believe that establishing the effectiveness of CTOs while they are in place is required prior to measuring any longer-term effects.

### Kisely et al's systematic review

Benefits following community treatment orders have an inverse relationship with rates of use: a meta-analysis and meta-regression by Kisely et al in a recent issue of the *BJPsych Open* is important because it provides clues on which patient groups may benefit from compulsory treatment.^10^ CTO use in Australasia was examined to evaluate whether factors associated with CTO use or subsequent outcomes vary according to rates of use. Kisely et al confirm the well-established finding that CTO use varies substantially between regions. They also confirm that CTO use is increased for single males not engaged in work or study and Australians with culturally and linguistically diverse (CALD) backgrounds. Their meta-regression reported that CTO recipients in jurisdictions with higher rates of CTO use were more likely to include females and diagnoses other than non-affective psychoses. High-use jurisdictions were less likely to show reductions in readmission rates or bed-days.

We think that the associations with high-use jurisdictions are intriguing. In our work evaluating associations with CTO use, we reported distinct outcome signatures according to diagnosis. All diagnostic groups received more community care and psychiatric medication, including intramuscular antipsychotic medication, during CTOs, but less frequent admissions and reduced bed-nights per year on CTOs only occurred for patients with psychotic disorders.^8,9^ The opposite occurred for other diagnostic groups. In our research, males but not females showed a significant reduction in admissions when on CTOs.^8^ We also reported that mortality rates were higher during CTOs compared with non-CTO periods.^7^ This finding diverged from other studies that compared mortality rates following CTO initiation with matched controls. The divergent finding may relate to the choice of comparator in CTO studies. It is feasible that CTO recipients have lower rates of mortality compared with matched non-CTO recipients and higher rates of mortality during CTOs compared with non-CTO periods. We regard these findings to be informative about the impacts of CTOs while they are in place. However, these were ‘real-world’ studies and further inferences, such as recommending more extensive use of CTOs for psychotic disorders because of the associations reported, should not be drawn from our studies.

Much of the previous research has focused on evaluating whether CTOs are associated with improved outcomes. The review by Kisely et al and our research suggest that reframing the research question is helpful. CTOs have different outcome signatures according to diagnosis and perhaps other demographic variables. Our research supported the use of CTOs to reduce admissions for people with psychotic disorders. For people with other diagnoses, it is possible that increased admissions on CTOs may be regarded favourably (for example, to provide electroconvulsive therapy for depression or containment for mania); however, some would regard this as a negative outcome. We suggest that clinicians integrate likely outcomes associated with CTOs according to diagnosis to inform conversations with patients and family.

We also think that the marked variation in CTO use confirmed by Kisely et al is of interest. Although this finding is well-recognised, the reasons underpinning variation are not well-understood. We have completed an analysis of regional variation in CTO use in New Zealand.^11^ This analysis controlled for underlying factors known to be associated with CTO use (age, gender, ethnicity and deprivation) to determine whether these factors explained the extent of variation. We concluded that sociodemographic variance between regions did not explain the wide range of CTO utilisation in New Zealand and that institutional culture and practice differences accounted for the variation in CTO use.

## Uniformity of use, legislative change and alternatives to CTOs

The medico-legal thresholds for CTO use should be applied consistently in the absence of legislative change. For equity reasons, we believe that rates of CTO use should not differ substantially between regions. Furthermore, the associations reported by Kisely et al suggest that high-use jurisdictions are less likely to show reductions in admissions associated with CTOs. This suggests that variation in CTO usage should be addressed because of differing outcomes according to use. We therefore suggest that regional variation in CTO use be a focus for jurisdictions using CTOs and that greater uniformity of CTO use is desirable. It is possible that workshops providing education in this area and cross-dissemination of clinical and legal staff would be beneficial.

The traditional criteria for CTO use are based on risk of harm to self or others. The research referred to above evaluates outcomes for these CTOs. There are concerns that risk-based mental health legislation is discriminatory and should be replaced by capacity-based legislation.^12^ There is also an interest in alternative forms of decision-making, such as advanced directives made prior to periods of unwellness.^13^ Given the controversial nature of CTOs, we support any efforts to ensure that alternatives to CTOs are explored. There is evidence that clinicians adapt to changing legislation and patients with the most severe illnesses (associated with risks or lack of capacity) continue to be treated compulsorily despite legislative change.^14^ However, we also believe that recent research, such as Kisely et al's excellent analysis,^10^ is assisting the field by informing which outcomes different patient groups are likely to experience on CTOs and that this will improve care for patients and families.

## Further research

We believe these recent studies suggest fruitful avenues for further research. We recommend that other researchers evaluate CTO outcomes according to diagnosis and in other subgroups, such as CALD communities, to see whether the associations reported in new Zealand are observed elsewhere. We also suggest further research to explore the impact of institutional practice and variation in CTO rates as a determinant of CTO outcomes. Finally, the changing nature of compulsory interventions require evaluation ideally undertaken in partnership with patients and families.

## Data Availability

Data availability is not applicable to this article as no new data were created or analysed in this study.
